# Prevalence of Hypertension in Young Athletes: A Community-Based Screening Analysis

**DOI:** 10.1016/j.jacadv.2025.102472

**Published:** 2025-12-18

**Authors:** Aneeq Malik, Jaipal Virdi, Timothy Le, Andre Sanavi, Shonit Nandakumar, Boback Ziaeian, Kyle Mefferd, Sasha Gladkikh, Jeffrey J. Hsu

**Affiliations:** a Department of Cardiology, Riverside Community Hospital, Riverside, California, USA; b Saving Hearts Foundation, Los Angeles, USA; c Division of Cardiology, Department of Medicine, David Geffen School of Medicine at UCLA, Los Angeles, USA; d Division of Cardiology, Department of Medicine, Veterans Affairs Greater Los Angeles Healthcare System and UCLA, Los Angeles, USA

**Keywords:** adolescent health, blood pressure screening, body mass index, cardiovascular risk, preparticipation evaluation, socioeconomic disparities

## Abstract

**Background:**

Although athletes are often perceived as low risk for cardiovascular disease, emerging evidence suggests they may still experience elevated blood pressure (BP). The prevalence and determinants of hypertension (HTN) in young athletes remain underexplored.

**Objectives:**

The objectives of the study were to determine the prevalence of elevated BP in a community-based cohort of young individuals, compare BP categories between athletes and nonathletes, and identify demographic, socioeconomic, environmental, and sport-related factors associated with HTN.

**Methods:**

We conducted a retrospective analysis of 1,987 individuals aged 9 to 35 years who attended free community cardiovascular screenings from 2016 to 2024. BP was measured using standardized protocols and classified per 2017 American Heart Association/American College of Cardiology guidelines. Multivariable ordinal logistic regression evaluated associations between HTN category and athlete status, sex, age, body mass index, socioeconomic status, air pollution exposure (particulate matter <2.5 μm), and environmental burden (CalEnviroScreen score). Athletes were additionally stratified by the isometric load of reported sports.

**Results:**

The median age was 15.7 years and 47.9% were females. Overall, 22.7% were classified as pre-HTN, 12.0% as stage I HTN, and 6.6% as stage II HTN. HTN prevalence did not differ significantly between athletes and nonathletes (*P* = 0.17). Higher BP category was associated with male sex, increased age, elevated body mass index, and lower socioeconomic status. Neither particulate matter <2.5 μm nor environmental burden predicted HTN. No significant difference in HTN prevalence was found across sport types with varying isometric loads.

**Conclusions:**

Elevated BP is common in young individuals, including athletes. These findings underscore the importance of BP screening in youth, regardless of athletic status, and highlight the influence of demographic and socioeconomic risk factors.

Athletes often epitomize peak physical fitness, health, and wellness. Rigorous training regimens and high levels of physical activity are widely accepted to confer cardiovascular benefits. However, emerging evidence suggests that athletes may still be vulnerable to cardiovascular risks and diseases, such as elevated blood pressure (BP), that can remain undetected due to their perceived overall health. Understanding the incidence of elevated BP in athletes is crucial, as hypertension (HTN), if undiagnosed or untreated, can pose long-term health risks even to those engaged in regular physical activity.

A retrospective analysis conducted by the Stanford Cardiology Program on over 2,500 athletes aged 13 to 35 years revealed that 35% had elevated BP consistent with a diagnosis of HTN.^1^ This finding is particularly significant given the presumed fitness and young age of the participants, challenging the conventional belief that athletes are universally protected from such cardiovascular risk factors. The prevalence of elevated BP in this population demonstrates the need for thorough cardiovascular assessments, even in individuals considered to be at peak fitness.

Preparticipation cardiovascular screening (PPCS) plays a critical role in identifying underlying cardiovascular issues, including elevated BP that could put athletes at risk. PPCS, which generally includes a medical history and physical examination, aims to detect conditions that may predispose athletes to cardiovascular complications during sports.^2^^,^^3^ Although PPCS has historically focused on identifying high-risk athletes for sudden cardiac death, elevated BP remains an important, yet often underappreciated, marker of cardiovascular health that should be addressed in these screenings, as elevated BP in young athletes increases the risk of developing pathological cardiovascular remodeling later in life.^4^

Community-based heart screening events offer an opportunity to assess the cardiovascular health of large populations, including both athletes and nonathletes. These screenings allow for comparative analyses between these groups and enable the identification of demographic and anthropometric factors, such as age, gender, and body composition, that may influence BP.

Using data from a community-based heart screening organization, this study aims to analyze the: 1) prevalence of elevated BP in young adults; 2) compare the prevalence between athletes and nonathletes; 3) analyze the difference in HTN rates between sports; and 4) determine risk factors contributing to elevated BP in these populations. By doing so, we hope to contribute to a better understanding of cardiovascular health in young athletes and provide insights to refine screening protocols and preventive strategies.

## Methods

### Study design

This study employed a retrospective analysis of data collected from a community-based health screening program in the Los Angeles area. Screenings were conducted between September 2016 and May 2024, with a temporary pause in activities from March 2020 through September 2021. A total of *25 community-based screening events* were held across *25 separate venues*, consisting primarily of *local high schools and community centers*. Although the heightened risk of sudden cardiac arrest in athletes was emphasized in educational messaging surrounding these events, there was no targeted advertisement or recruitment specifically directed toward athletes. Participation was open to the public, allowing inclusion of both athletes and nonathletes from the surrounding communities. Participation in the program was free and targeted primarily toward athletes, although individuals who did not participate in sports were also allowed to take part. Sport participation was self-reported, with no distinctions based on the frequency of participation or the level of competitiveness. Participants were eligible for inclusion if they were between 9 and 35 years of age and had a valid BP reading. Those who fell outside the specified age range or had a prior diagnosis of HTN were excluded from the study.

This study was reviewed by HCA Healthcare’s C.A.R.R.I.E. (Centralized Algorithms for Research Rules on IRB Exemption) system and was determined to be exempt from Institutional Review Board (IRB) oversight under applicable federal regulations and institutional policy (reference ID: 2024-712). The exemption was granted on July 17, 2024. The study was found to meet the criteria for exemption due to sufficient protections in place and the use of deidentified data. All research activities were conducted in accordance with institutional policies and the ethical principles outlined in the Belmont Report. Specific zip code data cannot be disclosed due to IRB restrictions designed to preserve deidentification and participant privacy.

### Preparticipation evaluation

PPCS were conducted by trained volunteers associated with the community-based program at local high schools. All volunteers had undergone prior training in standardized BP measurement techniques. The screening protocol followed the American Heart Association (AHA)’s 14-element cardiovascular screening model and included the collection of resting BP and measurements of height and weight. Ethnicity was self-reported by participants.^5^ All final determinations of PPCS results were conducted by an experienced cardiologist.

### Measurement and classification of BP

Resting BP was obtained at the time of the initial screening using a standardized protocol. An automated oscillometric cuff was used, with the cuff positioned at the level of the heart and sized appropriately for each participant. Measurements were taken while the participant was seated with their forearm resting flat on a supportive surface. For individuals with an initial BP reading of ≥140/90 mm Hg, a second measurement was taken on the opposing arm following a brief resting period. If the second reading also exceeded 140/90 mm Hg, participants were advised to follow up with their primary care physician. BP was classified according to the 2017 American College of Cardiology (ACC)/AHA guidelines for all participants, which are consistent with the most contemporary guidelines, regardless of age.^6^^,^^7^ Although these guidelines are formally applicable to individuals aged 13 years and older, they are largely consistent with the 2017 American Academy of Pediatrics recommendations for younger children, which incorporate age-, sex-, and height-specific percentiles. Given the close alignment between the 2 systems, applying the ACC/AHA thresholds across all age groups ensured analytical consistency without meaningfully affecting classification.^8^

### Socioeconomic status

Socioeconomic status (SES) was determined based on the average household income associated with the zip code of each participant’s residence. Data on average household income were obtained from the U.S. Census Bureau.^9^ SES was categorized into 3 levels: low, moderate, and high. Participants were classified as low SES if the average income for their zip code was below the 33rd percentile of average California household income, moderate SES if the average income was between the 33rd and 66th percentiles, and high SES if the average income was above the 66th percentile.

### Neighborhood burden

Neighborhood burden was assessed using the CalEnviroScreen (CES) 4.0 score, a statewide index developed by California's Office of Environmental Health Hazard Assessment. The CES integrates 13 pollution indicators (including diesel traffic, pesticide use, and hazardous-waste proximity) with 8 population-vulnerability indicators (such as linguistic isolation, unemployment, and asthma emergency department visits). These indicators are averaged over the most recent 3- or 5-year period, then the pollution and vulnerability domain scores are multiplied together and scaled to express each census tract as a 0 to 100 percentile; higher percentiles indicate greater combined environmental and social burden. Participant CES percentiles were determined by linking self-reported zip codes to corresponding tract-level data. This cumulative approach to measuring environmental burden has established precedent as a predictor of health outcomes across various conditions.^10^^,^^11^

### Air pollution

Air pollution was defined as a measure of ambient PM_2.5_ concentrations (particulate matter ≤2.5 μm in diameter) and was obtained from the California Air Resources Board’s ground-based air monitoring network. In zip codes lacking monitors, data were received from satellite-derived remote-sensing models. Continuous measurements from fixed stations and satellite estimates were aggregated to annual mean concentrations for each zip code, then linked to participants via their self-reported residence. Both short and long-term exposures to elevated PM_2.5_ levels have been associated with higher BP and an increased risk of HTN.^12^^,^^13^

### Sport subgroup analysis

To analyze the relationship between sport type and HTN prevalence, we grouped sports based on their isometric load.^14^ Isometric load refers to the degree of static muscle contraction required during activity. Participants who reported engaging in sport were categorized into 3 classes.

1. Class I sports (low isometric load): golf, baseball/softball, volleyball, soccer, tennis
2. Class II sports (moderate isometric load): football, track/cross country, swimming/diving, hockey, basketball, lacrosse
3. Class III sports (high isometric load): dance/gymnastics/cheer, weightlifting, martial arts, wrestling

Participants who reported engaging in multiple sports or sports that did not fit these classifications were excluded from this subgroup analysis.

### Statistical analysis

To compare the distribution of HTN categories between athletes and nonathletes, an unadjusted cumulative odds ordinal logistic regression was conducted, ensuring methodological consistency with the adjusted multivariable model. In addition, a cumulative odds ordinal logistic regression model was employed to evaluate the effects of sex, race, sport participation, SES, air pollution, neighborhood burden, and body mass index (BMI) on HTN category membership. The proportional odds assumption was assessed using a full likelihood ratio test, which compared the fitted model with a model that allowed for varying location parameters.

A chi-square test of homogeneity was conducted to determine whether the distribution of participants in stage I HTN or stage II HTN categories differed significantly across the 3 isometric load categories (low, moderate, and high). The adequate sample size for the chi-square test was determined according to Cochran, ensuring no cell had an expected frequency of <5.^15^ Statistical significance was set at *P* < 0.05.

## Results

### Screening cohort

**Figure 1:**
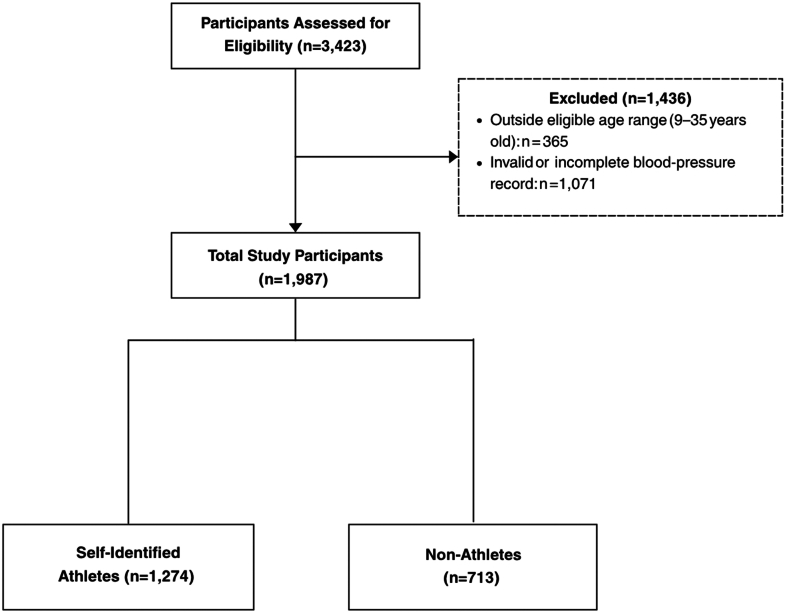
**Enrollment and Exclusion for Youth Hypertension Study** Flowchart depicting participant selection. Of the 3,423 individuals assessed, 1,436 were excluded due to age ineligibility or invalid blood pressure data, leaving 1,987 participants. These participants were then stratified based on questionnaire responses to sport participation as self-identified athletes (n = 1,274) and nonathletes (n = 713).

A total of 3,423 participants underwent community-based health screenings between September 2016 and May 2024. After applying the inclusion and exclusion criteria, 1,987 participants were included in the final analysis (Figure 1). Participants ranged in age from 9.5 to 34.2 years, with a mean age of 16.2 years and a median age of 15.7 years. Of the included participants, 1,027 (51.7%) were males, 952 (47.9%) were females, and 8 (0.4%) identified as other.

Among the 1,987 participants analyzed, 1,274 (64.1%) reported engaging in sport, whereas 713 (35.9%) did not. The most commonly reported sports among athletes were soccer (9.50%), track and cross country (7.14%), basketball (6.28%), swimming/diving (5.33%), and dance/gymnastics/cheer (5.18%). Other frequently reported sports included football (4.88%), tennis (3.22%), and baseball/softball (3.02%).

SES distribution showed that 334 (16.8%) were from high-income areas, 1,415 (71.2%) from moderate-income areas, and 179 (9.0%) from low-income areas, with SES data unavailable for 59 participants (3.0%). Ethnic distribution revealed that 840 (42.3%) participants identified as Caucasian, 474 (23.9%) as Hispanic, and 387 (19.5%) as Asian, with 286 (14.4%) as smaller representations from other groups (Table 1).

**Table 1:** Population Summary by Athletic Status

	Athlete	Nonathlete	Total
Overall			
Sample size (N)	1,274	713	1987
Demographics			
Age (y)	15.64 ± 2.63	17.11 ± 3.82	16.17 ± 3.19
BMI (kg/m^2^)	21.75 ± 4.21	22.64 ± 4.98	22.07 ± 4.52
BMI category			
Underweight	245 (19.2%)	111 (15.6%)	356 (17.9%)
Normal	726 (57.0%)	387 (54.3%)	1,113 (56.0%)
Overweight	159 (12.5%)	105 (14.7%)	264 (13.3%)
Obese	54 (4.2%)	58 (8.1%)	112 (5.6%)
Gender			
Female	568 (44.6%)	384 (53.9%)	952 (47.9%)
Male	702 (55.1%)	325 (45.6%)	1,027 (51.7%)
Other	4 (0.3%)	4 (0.6%)	8 (0.4%)
Race/ethnicity			
Asian	206 (16.2%)	181 (25.4%)	387 (19.5%)
Caucasian	569 (44.7%)	271 (38.0%)	840 (42.3%)
Hispanic	305 (23.9%)	169 (23.7%)	474 (23.9%)
Other	194 (15.2%)	92 (12.9%)	286 (14.4%)
Socioeconomic status			
High	208 (16.3%)	126 (17.7%)	334 (16.8%)
Moderate	960 (75.4%)	455 (63.8%)	1,415 (71.2%)
Low	85 (6.7%)	94 (13.2%)	179 (9.0%)
Blood pressure category			
Normal	763 (59.9%)	403 (56.5%)	1,166 (58.7%)
Pre-HTN	280 (22.0%)	171 (24.0%)	451 (22.7%)
Stage I HTN	147 (11.5%)	91 (12.8%)	238 (12.0%)
Stage II HTN	84 (6.6%)	48 (6.7%)	132 (6.6%)
Environmental measurements			
CES score	28.55 ± 10.60	29.67 ± 12.36	28.93 ± 11.22
PM_2.5_ (μg/m^3^)	10.39 ± 1.21	10.45 ± 1.19	10.41 ± 1.20

Values are mean ± SD or n (%). This table presents demographic, clinical, and environmental characteristics for both the overall population and when it is stratified by athletic status.

BMI = body mass index; CES = CalEnviroScreen; HTN = hypertension; PM_2.5_ = particulate matter <2.5 μm.

### HTN classification

**Figure 2:**
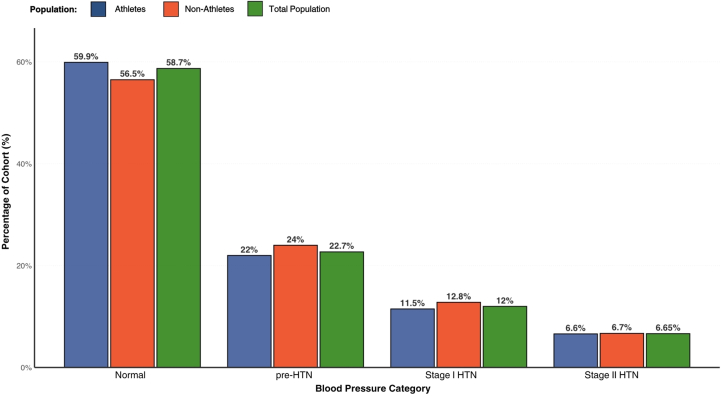
**Distribution of Hypertension Classes by Sports Participation** Bar chart showing the percentage distribution of blood pressure. Bar chart showing the percentage distribution of blood pressure categories (normal, pre-HTN, stage I HTN, stage II HTN) among athletes, nonathletes, and the total population in our data set. HTN = hypertension.

Of the 1,987 that met the inclusion criteria, BP classification showed that 1,166 (58.7%) participants fell within the normal range, whereas 451 (22.7%) were categorized as pre-HTN, 238 (12.0%) as stage I HTN, and 132 (6.6%) as stage II HTN. A cumulative odds *ordinal logistic regression* with proportional odds was performed to examine the association between *athlete status (athlete vs non-athlete)* and HTN category. The proportional odds assumption was met, as verified by a full likelihood ratio test comparing the fitted model to one with varying location parameters (chi-square [2] = 0.447, *P* = 0.800). The deviance goodness-of-fit test indicated that the model adequately fit the observed data (chi-square [2] = 0.447, *P* = 0.224). However, the model did not significantly predict HTN category beyond the intercept-only model (chi-square [1] = 38.982, *P* = 0.177), indicating that *athlete status was not a significant predictor of HTN classification* (Figure 2).

**Figure 3:**
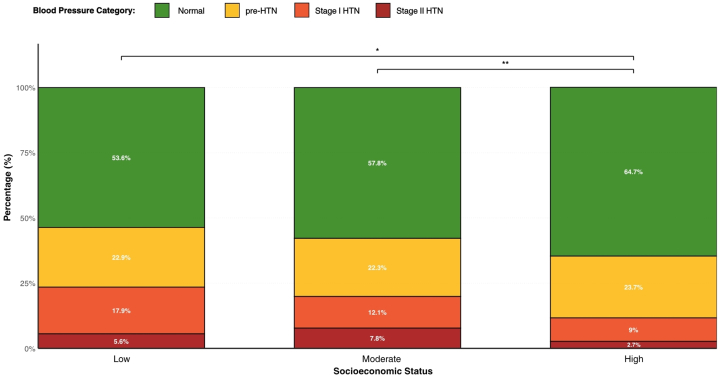
**Distribution of Hypertension Class by Socioeconomic Status** Stacked bar chart displaying the percentage distribution of blood pressure categories (normal, Prehypertension, stage I hypertension, stage II hypertension) across low, moderate, and high socioeconomic status groups. Statistical comparisons between socioeconomic groups were performed using chi-square tests with Bonferroni correction for multiple comparisons. Asterisks indicate statistically significant differences: ∗*P* < 0.05, ∗∗*P* < 0.01. Abbreviation as in Figure 2.

For participants with available data, a Pearson’s chi-square test of independence was conducted to examine the association between SES and BP category distribution (Figure 3). The analysis revealed a statistically significant difference in distribution across SES groups (chi-square = 21.897, *P* = 0.001). Post hoc pairwise comparisons using Bonferroni-corrected chi-square tests identified specific differences between the high SES group and both the low (*P*_adj_ = 0.016) and moderate SES groups (*P*_adj_ = 0.005). No significant difference was observed between the low and moderate SES groups (*P*_adj_ = 0.372).

### Predictors of elevated BP

**Figure 4:**
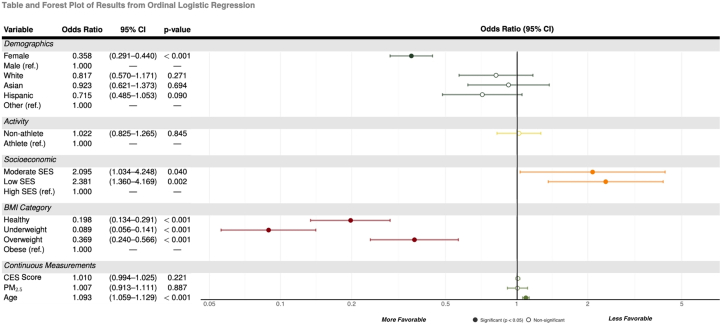
**Predictors of Hypertension Class** Forest plot and table show OR (95% CI) from ordinal logistic regression for demographic, socioeconomic, activity, BMI, and continuous variables. ORs >1.00 indicate higher odds of belonging to a higher hypertension class; <1.00 indicates lower odds. Significant predictors (*P* < 0.05) are shown in dark markers. BMI = body mass index; CES = CalEnviroScreen; PM_2.5_ = particulate matter <2.5 μm; SES = socioeconomic status.

SES, age, and BMI were significant predictors of HTN category membership (Figure 4). Women were 0.358 times less likely than men to be in a higher HTN category (95% CI: 0.291-0.440; chi-square [1] = 95.212; *P* < 0.001). Participants from low- and moderate-income areas had a 2.381 and 2.095 increase, respectively, in the log odds of being in a higher HTN category compared to those from high-income areas (95% CI: 1.360-4.169; chi-square [1] = 9.215; *P* = 0.002 and 95% CI: 1.034-4.248; chi-square [1] = 4.209; *P* = 0.040). Age was also a significant predictor of belonging to a higher HTN category (OR: 1.093; 95% CI: 1.059-1.129; chi-square [1] = 30.145; *P* < 0.001). Predicting significantly lower odds of belonging to a higher HTN category.

BMI also showed a strong association with the HTN category, with lower BMI categories predicting significantly lower odds of belonging to a higher HTN category. When compared to the highest BMI category, the overweight BMI category (OR: 0.369; 95% CI: 0.369-0.240; chi-square [1] = 20.757; *P* < 0.001), the healthy BMI category (OR: 0.198; 95% CI: 0.134-0.291; chi-square [1] = 67.342; *P* < 0.001), and the underweight BMI category (OR: 0.089; 95% CI: 0.056-0.141; chi-square [1] = 107.356; *P* < 0.001) were significantly predicted to be in a lower HTN category.

Higher scores of PM_2.5_ and CES.4.0 did not significantly predict falling into a higher HTN category. Race was also not a significant predictor of HTN category.

As a sensitivity analysis, a *multinomial logistic regression* was also performed to test whether relaxing the proportional odds assumption altered model performance. The results were consistent with the ordinal logistic regression, with age, sex, SES, and BMI remaining significant predictors of HTN classification (see Supplemental Table 1). As an additional sensitivity analysis, a stratified model comparing adults and children was performed, which demonstrated consistent findings, with children less likely to fall into higher HTN categories.

### Sport subgroup analysis

Of the 1,274 participants who reported engaging in sports, 1,122 were categorized into 1 of 3 isometric load classes: 428 (38.1%) in class I (low isometric load), 531 (47.3%) in class II (moderate isometric load), and 163 (14.5%) in class III (high isometric load). The remaining 152 participants who engaged in multiple or unclassified sports were excluded from this analysis.

In class I (low isometric load), 12.9% of participants were classified as stage I HTN and 6.8% as stage II HTN. In class II (moderate isometric load), 11.1% were classified as stage I HTN and 7.2% as stage II HTN. In class III (high isometric load), 8.6% were classified as stage I HTN and 4.3% as stage II HTN. The 2 multinomial probability distributions were equal in the population, chi-square [1] = 0.529, *P* = 0.767, indicating no significant difference in the distribution of HTN stages across the 3 isometric load classes.

## Discussion

The findings from this study underscore the significant prevalence of elevated BP among a young population, with nearly 1 in 5 participants exhibiting readings consistent with either stage I or stage II HTN. This prevalence is consistent with results found in prior studies. For example, Baggish et al.^16^ reported that 19.2% of National Collegiate Athletic Association Division I football players had HTN and 61.9% had pre-HTN with the significantly higher pre-HTN rate likely reflected by a more specific athlete population. Kropa et al.^17^ examined urban high school athletes and found that 14.8% had stage I or II HTN, which is comparable to the 18.6% prevalence we observed in the present study, especially considering the socioeconomically diverse and racially heterogeneous setting of our cohort. Lastly, National surveillance data from National Health and Nutrition Examination Study showed lower rates among U.S. youth overall (10% HTN, 21% borderline), although these samples skew younger and do not focus specifically on athletic or underserved populations.^18^ Our study findings reinforces these results among a broader demographic encompassing both young athletes across various sports and those who do not participate in sport.

Interestingly, the rates of stage I and stage II HTN were similar between those who participated in sports and those who did not. This finding suggests that participation in sports, traditionally considered a protective factor against HTN, may not necessarily confer the expected benefits in this population.^19^, ^20^, ^21^ It is possible that other risk factors unique to adolescence and young adults—particularly those from urban, socioeconomically diverse populations—such as poor dietary habits, psychological stress, or mental health challenges, might mitigate the protective effects of regular physical activity. Future research should explore these potential influences to better understand their contributions to BP elevation in young athletes.

Beyond the prevalence patterns, it is important to consider the long-term physiological implications of elevated BP in young individuals. Chronic elevations in BP can lead to increased left ventricular afterload, vascular remodeling, and endothelial dysfunction, laying a foundation for long-term cardiovascular disease. These effects are particularly concerning when they begin in youth. In young adults and athletes, prior studies have demonstrated that even modest BP elevations may initiate deleterious cardiovascular changes.^22^ For instance, Kim et al.^4^ found that even short-term elevations in BP among college athletes led to increases in left ventricular mass and reduced diastolic function, indicating the emergence of a maladaptive cardiovascular phenotype. Similarly, Baggish et al.^16^ observed concentric hypertrophy and abnormal diastolic filling patterns in young athletes with HTN. These findings suggest that athletic conditioning may not fully buffer the negative cardiovascular effects of elevated BP. Importantly, elevated BP in adolescence and young adulthood has been shown to track into later life, increasing the risk for sustained HTN, cardiovascular disease, and premature mortality.^18^^,^^23^ These insights underscore the need to identify and address elevated BP early—even in those presumed to be low risk, such as active youth.

The results of the sport subgroup analysis revealed no significant association between the isometric load of sports and the prevalence of stage I or stage II HTN in young athletes. Despite the theoretical expectation that sports with higher isometric demands, such as weightlifting and wrestling, might contribute to higher rates of elevated BP, the findings did not support this hypothesis. The distribution of HTN was remarkably consistent across low, moderate, and high isometric load categories, suggesting that the type of muscular effort required in these sports may not be a primary factor influencing HTN risk. This consistency may reflect the complex interaction of other factors such as body composition, training volume, diet, and psychological stress, which were not fully accounted for in this analysis. In addition, the self-reported nature of sport participation and the lack of differentiation between levels of competition or training intensity could have limited the sensitivity of the analysis. Furthermore, positional variability within certain sports, such as football or basketball, may contribute to differing cardiovascular demands, which were not captured in this study.

Notably, lower SES was strongly associated with higher rates of stage I and stage II HTN. Participants from low-income areas were significantly more likely to exhibit elevated BP compared to their peers from high-income areas. This finding likely reflects the multifaceted challenges faced by individuals of lower SES, including increased susceptibility to unhealthy dietary patterns, higher levels of financial and psychosocial stress, limited opportunities for regular physical activity, and barriers to adequate mental health support.

Traditional risk factors for HTN, such as male sex, age and obesity, were also confirmed as significant predictors in this analysis, aligning with findings from prior research.^17^^,^^18^ Males were significantly more likely than females to belong to higher BP categories, and participants with obesity demonstrated markedly increased odds of elevated BP compared to those with lower body mass indices. Specifically, being a healthy weight can reduce the risk for being in a higher HTN category by nearly 80%. These results reinforce the established role of these risk factors in the development of HTN and emphasize the need for interventions targeting weight management and gender-specific health education in this age group.

Surprisingly, neither ambient PM_2.5_ concentration nor the cumulative CES score predicted HTN health outcomes. This lack of association may reflect several factors: the relatively short exposure histories and young age of the cohort, which limit the dose needed for potential cardiovascular effects; substantial within-zip-code variability that can mask individual-level exposures; and the still-evolving evidence linking these hazards to HTN in younger populations. Together, these considerations suggest that finer-scale exposure metrics and longer follow-up will be needed to clarify these relationships.

Other potential contributors include poor sleep hygiene and the consumption of stimulants such as energy drinks, which are often prevalent in this demographic but were not assessed in this study. Nevertheless, the high prevalence of elevated BP in this young population calls attention to the need for targeted public health interventions that address the social determinants of health contributing to HTN in underserved communities.

Recent large-scale opportunistic screening initiatives further emphasize the importance of early detection in young and otherwise low-risk populations. For example, Tan et al.^24^ demonstrated the feasibility of “pop-up” cardiovascular screenings conducted in over 76,000 individuals across community pharmacies and a major sporting event, identifying that nearly 70% of participants had at least 1 uncontrolled cardiovascular risk factor, including 37% with elevated BP. Notably, more than half of those with elevated BP had not had a BP check in the prior year, and over 80% were not receiving antihypertensive therapy, underscoring both the underdiagnosis and undertreatment of HTN in the community. These findings align with our observation of substantial HTN prevalence among youth, and together they highlight the potential value of opportunistic screening in nontraditional settings for improving detection, linkage to care, and ultimately long-term cardiovascular outcomes.

### Study Limitations

This study has several limitations. First, a clinical diagnosis of HTN is based on BP measurements from 2 different occasions, precluding the ability to diagnose HTN in participants in our study. In addition, BP measurements were taken during a community-based event, which may not fully align with the standardized protocols recommended by the AHA/ACC. Factors such as participant stress, environmental noise, or improper posture during measurement could have introduced variability. In addition, single-point BP readings do not account for the possibility of white coat HTN or other transient conditions that may elevate BP readings temporarily. Confirmatory testing performed in an outpatient setting under controlled conditions would be necessary to validate the findings.

Second, the self-reported nature of sport participation and demographic data, such as SES and ethnicity, may introduce reporting bias or misclassification. In addition, there was no distinction made between the level of competition of sport or the frequency of participation. SES was derived using the zip code of each screening location, as individual addresses were unavailable due to IRB deidentification requirements. Although we acknowledge that substantial heterogeneity may exist within zip codes—particularly in an urban area such as Los Angeles—this represented the most granular and accurate geographic proxy available given the data and privacy constraints.

In addition, a subset of early screening events relied on paper-based data collection and, in 1 case, equipment malfunction, which contributed to missing or invalid BP data. A small number of duplicate records were also identified and removed during data cleaning to ensure each participant was represented only once. Despite these limitations, the large sample size and standardized data collection protocols used in later events provide robust insights into BP patterns among young individuals in diverse community settings.

## Conclusions

The adoption of the 2017 ACC/AHA guidelines for BP classification revealed a substantial proportion of this young population at risk of HTN (Central Illustration). Recent efforts have been made by the ACC to highlight the specific challenges in managing the cardiovascular health of young athletes.^25^ Our study highlights the necessity of comprehensive screening and early intervention strategies, particularly for young athletes who might otherwise be perceived as low risk. Such interventions should focus on lifestyle modifications, including proper nutrition, stress management, mental health support, and fostering healthier sleep habits. By addressing these modifiable risk factors, it may be possible to mitigate the long-term cardiovascular risks associated with elevated BP in this population.

Perspectives **COMPETENCY IN MEDICAL KNOWLEDGE:** This study contributes to the medical knowledge and systems-based practice competency domains by challenging the assumption that young athletes are universally protected from cardiovascular risk. Clinicians and trainees will benefit from an enhanced understanding of the importance of routine BP assessment in adolescents and young adults—regardless of athletic participation. The findings support broader implementation of BP screening during preparticipation evaluations (PPCS) and routine care, particularly in populations at risk due to socioeconomic disparities or elevated BMI. The study also informs practice-based learning, encouraging providers to reconsider existing protocols and identify gaps in risk stratification for hypertensive youth, including athletes.

**TRANSLATIONAL OUTLOOK:** Although community-based cardiovascular screening offers a scalable method for early detection of HTN, there remain significant barriers to translation into consistent clinical practice. These include the lack of standardized follow-up pathways after elevated BP detection, limited awareness of risk among youth and their caregivers, and variability in preparticipation screening protocols. Future research should focus on longitudinal studies to validate these findings across more diverse populations and examine causal mechanisms, particularly the role of psychosocial stress, dietary patterns, and stimulant use. In addition, translational efforts should prioritize integrating cardiovascular risk assessments into school-based or primary care settings, leveraging digital tools and mobile health technology to improve screening reach and follow-up care.

**Central Illustration:**
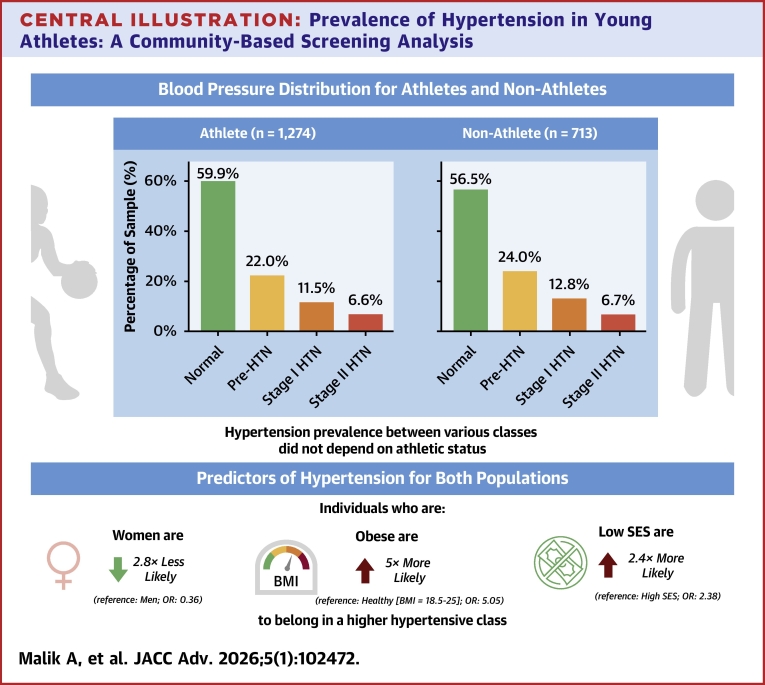
**Prevalence of Hypertension in Young Athletes: A Community-Based Screening Analysis** Distribution of blood pressure categories (normal, prehypertension, stage I, and sage II hypertension) among self-reported athletes (n = 1,274) and nonathletes (n = 713) from community-based screenings. HTN prevalence did not differ significantly by athletic status. In multivariable ordinal logistic regression, female sex was protective (OR: 0.36), whereas obesity (OR: 0.05) and low socioeconomic status (OR: 2.38) were major risk factors. Additional predictors followed similar directional trends. These findings indicate that elevated blood pressure is common among youth and young adults regardless of athletic participation and is influenced primarily by demographic and socioeconomic determinants. BMI = body mass index; HTN = hypertension; SES = socioeconomic status.

## Funding support and author disclosures

This research was supported in part by HCA Healthcare and/or an HCA Healthcare affiliated entity. The views expressed in this publication represent those of the authors and do not necessarily represent the official views of HCA Healthcare or any of its affiliated entities. The authors have reported that they have no relationships relevant to the contents of this paper to disclose.
