# Supplementary material for: Prevalence of Hypertension in Young Athletes: A Community-Based Screening Analysis
Source: JACC Adv. 2025 Dec 18;5(1):102472. doi: 10.1016/j.jacadv.2025.102472 (PMC12775954; doi:10.1016/j.jacadv.2025.102472)
Supplement: Supplemental_File [file mmc1.pdf]

Supplemental Material

Supplemental Table S1

Multinomial Logistic Regression Results for Blood Pressure Categories

HTN Class	Variable	Odds Ratio	95% CI	P-Value
Pre-HTN (ref. Normal)				
	Age	1.091	(1.045, 1.139)	0.055
	PM2.5	0.968	(0.855, 1.094)	0.600
	CES Score	1.006	(0.987, 1.026)	0.528
	Female	0.525	(0.409, 0.674)	0.000
	Male (ref.)	—	—	—
	Asian	1.027	(0.646, 1.633)	0.911
	Black	1.229	(0.741, 2.038)	0.425
	Hispanic	0.886	(0.421, 1.866)	0.751
	Other	1.006	(0.611, 1.658)	0.980
	White (ref.)	—	—	—
	Sport: No	1.091	(0.839, 1.419)	0.516
	Sport: Yes	—	—	—
	SES: Low	1.870	(0.933, 3.750)	0.078
	SES: Moderate	1.971	(0.827, 4.696)	0.126
	SES: High (ref.)	—	—	—
	Healthy	0.386	(0.212, 0.701)	0.002
	Underweight	0.226	(0.117, 0.434)	0.000
	Overweight	0.509	(0.264, 0.981)	0.044
	Obese (ref.)	—	—	—
Stage I HTN (ref. Normal)				
	Age	1.082	(1.025, 1.142)	0.004
	PM2.5	1.125	(0.954, 1.327)	0.160
	CES Score	1.001	(0.975, 1.027)	0.951
	Female	0.290	(0.204, 0.413)	0.000
	Male (ref.)	—	—	—
	Asian	0.825	(0.451, 1.511)	0.533
	Black	1.142	(0.594, 2.194)	0.690
	Hispanic	0.844	(0.343, 2.082)	0.714
	Other	0.736	(0.387, 1.399)	0.349
	White (ref.)	—	—	—
	Sport: No	1.091	(0.767, 1.551)	0.628
	Sport: Yes	—	—	—
	SES: Low	1.594	(0.647, 3.925)	0.311
	SES: Moderate	1.198	(0.372, 3.853)	0.762

	SES: High (ref.)	—	—	—
	Healthy	0.145	(0.078, 0.269)	0.000
	Underweight	0.055	(0.026, 0.119)	0.000
	Overweight	0.312	(0.158, 0.615)	0.001
	Obese (ref.)	—	—	—
Stage II HTN (ref. Normal)				
	Age	1.185	(1.115, 1.259)	0.000
	PM2.5	0.921	(0.753, 1.126)	0.422
	CES Score	1.032	(0.999, 1.066)	0.057
	Female	0.141	(0.082, 0.241)	0.000
	Male (ref.)	—	—	—
	Asian	0.623	(0.301, 1.288)	0.201
	Black	0.540	(0.231, 1.262)	0.155
	Hispanic	0.488	(0.157, 1.524)	0.217
	Other	0.450	(0.207, 0.981)	0.044
	White (ref.)	—	—	—
	Sport: No	0.869	(0.543, 1.390)	0.558
	Sport: Yes	—	—	—
	SES: Low	10.627	(2.846, 39.690)	0.000
	SES: Moderate	5.726	(1.077, 30.444)	0.041
	SES: High (ref.)	—	—	—
	Healthy	0.085	(0.042, 0.171)	0.000
	Underweight	0.012	(0.003, 0.044)	0.000
	Overweight	0.223	(0.104, 0.478)	0.000
	Obese (ref.)	—	—	—

S1 Table: Multinomial Logistic Regression Results for Blood Pressure Categories

Odds ratios, 95% confidence intervals, and p-values for predictors of blood pressure categories (Pre-HTN, Stage I HTN, and Stage II HTN) compared to Normal blood pressure.
